# Determination of Phosphorus in Soil by ICP-OES Using an Improved Standard Addition Method

**DOI:** 10.1155/2018/1324751

**Published:** 2018-09-13

**Authors:** Jing Yang, Jingwen Bai, Meiyu Liu, Yang Chen, Shoutong Wang, Qiyong Yang

**Affiliations:** ^1^Institute of Mountain Hazards and Environments, Chinese Academy of Sciences, Chengdu, Sichuan, China; ^2^Institute of Karst Geology Chinese Academy of Geological Sciences, Guilin, Guangxi, China

## Abstract

In this study, an improved standard addition method (ISAM) was developed for the determination of phosphorus in soil by ICP-OES based on the conventional standard addition method (CSAM) and calibration curve method (CCM). Certified standard soils were analyzed by the proposed ISAM method. The values obtained by ISAM method agreed with the certified values. Additionally, the results obtained by ISAM method were compared with those determined by the other two methods (CSAM and CCM). All the values obtained by the ISAM agreed with those from the other two methods. The detection limit, quantification limit, and recovery rate of each method were calculated, and the recovery rates of soil samples and the blank were all within the range of 90%–110%. Finally, the proposed method was applied to determine phosphorous in soil samples from Guangnan County, Yunnan Province, China, and the meadow soil from Qinghai-Tibet Plateau, China. The relative errors between the results from ISAM and CCM were all within 10%, and *t*-test showed that the results between ISAM and CCM had no significant difference (*P* > 0.05). Therefore, the proposed method overcame the matrix effect in some extent and was an acceptable method for the rapid and accurate batch analysis of P content in soil sample, especially batch samples with obvious matrix effect.

## 1. Introduction

Determination of total phosphorus (P) in soils is very important in agriculture as the amount of phosphorus is related to crop production. Recently, research on the biogeochemical cycle has become a significant focus in the fields of soil science, ecology, and geology. However, some of the research has focused on the phosphorus cycle [1–5]. Therefore, a reliable and rapid method is essential for the determination of phosphorus in soil.

Molybdenum blue method is the traditional technique for determination of phosphorus in soil [6–8]. This method has some disadvantages including complicated steps and procedures, being influenced by human operation, and low analytical efficiency. It is not suitable for the rapid and batch determination of soil samples. Inductively coupled plasma optical emission spectroscopy (ICP-OES) is an essential, modern analytical tool that has a high sensitivity and a broad linear range. In recent years, ICP-OES has been used in determination of phosphorous in geological samples [9], high-purity nickel [10], and biodiesel B100 from feedstock [11]. There are several quantitative methods used in ICP-OES mainly included calibration curve method (CCM) [12–15] and conventional standard addition method (CSAM) [16, 17]. The CCM is simple, but it is the susceptible to errors by fluctuations in operating conditions and the matrix effect. However, internal standard has been used to minimize errors caused by instrumental drift and to reduce chemical matrix effect [18–20]. CSAM is useful when the sample has obvious matrix effect and the matrix-matching method cannot be used. However, it is time-consuming and required a complex procedure.

Therefore, an improved standard addition method (ISAM), based on the CSAM and CCM, was proposed in this study for the determination of total phosphorus in soil. In this method, soil mixture solution was determined by the CSAM. The value of slope was calculated from the obtained calibration curve. All the samples were measured directly by ICP-OES for the determination of P, and the response signal values were obtained. Then, the total *P* value of each test sample could be calculated. Be similar to CSAM, the improved method overcomes the matrix effect. And be similar to CCM, ISAM was simple and rapid. So it is suitable for the rapid and accurate analysis of batch samples, especially the large number samples with obvious matrix effect. The difference between each analysis strategy is summarized in Table 1.

To verify the reliability of the novel method, 4 types of certified standard soils were analyzed, and the results were compared with those determined by the CCM and the CSAM. The values obtained were in close agreement with the certified values and the results from CCM and the CSAM. The detection limit and quantification limit were measured. Meanwhile, the proposed method was applied to determine phosphorous in soil samples from Guangnan County of Yunnan Province, China, and the meadow soil from Qinghai-Tibet Plateau, China.

## 2. Materials and Methods

### 2.1. Materials

Concentrated nitric acid (GR, guarantee reagent) and hydrofluoric acid (GR, guarantee reagent) were purchased from Sinopharm Chemical Reagent Co., Ltd., China. A stock standard solution of phosphorus (P) (1000 *μ*g/mL) was purchased from the National Center of Analysis and Testing for Nonferrous Metals and Electronic Materials, and a stock standard solution of rhenium (Re) (1000 *μ*g/mL) was purchased from Shanghai Macklin Biochemical Co., Ltd., China. Working standard solutions were freshly prepared from those stock solutions as required.

Four certified standard soils—GBW (E) 070041 (Institute of Soil Science, Chinese Academy of Science), GSS-5 and GSS-8 (Institute of Geophysical and Geochemical Exploration, Ministry of Geology and Mineral Resources), and GSS-14 (Institute of Geophysical and Geochemical Exploration, IGGE)—were obtained for the determination of total phosphorus. Soil samples were from Guangnan County, Yunnan Province, China, and Qinghai-Tibet Plateau, China. The soils were air-dried and passed through a 2 mm sieve, and subsamples were further ground to pass through a 0.25 mm sieve.

### 2.2. Apparatus

An Optima 8300 ICP-OES (PerkinElmer, USA) was applied for the analysis, and the instrumental specifications are given in Table 2. And an 18.2 MΩ deionized pure water treatment system (Millipore, USA) was used for preparing deionized water. A closed-vessel microwave digestion system (Mars 6, CEM, US) was applied to perform microwave-assisted digestion procedures for the soil samples. It is equipped with a 40-position rotor. A heating apparatus (BHW-09C, Shanghai Botong Chemical Technology Co. LTD, China) was employed to evaporate the residual acid.

### 2.3. Microwave Digestion

Approximately 0.15 g of a soil sample was weighed (with an accuracy of 0.0001 g) and placed in 50 mL Teflon vessel. Concentrated nitric acid (8 mL) and hydrofluoric acid (4 mL) were added. The closed vessels were introduced in a microwave oven-assisted sample digestion system. For complete digestion of the samples, a heating program comprising three steps was used. The program of the microwave dissolution is presented in Table 3. After cooling to room temperature, the digestion solution in Teflon vessel was evaporated to about 1 mL in a heating apparatus at 175°C. Evaporation was a necessary step since acid concentrations would have been too high for the ICP-OES. Careful operation was needed to avoid drying of the evaporation residues. Then, all solutions were transferred to a 100 mL volumetric flask and accurately diluted to the mark with deionized water. Run blanks with all the chemicals and process except the soil sample.

### 2.4. Preparation of Calibration Working Standards

#### 2.4.1. Calibration Working Standards for the ISAM

Soil mixture solution was obtained from the mixture of digested soil solutions.

A stock calibration standard of P (200 mg/L) was prepared by diluting 10 mL 1000 mg/L P calibration standard solution to 50 mL with deionized water. A stock calibration standard solution of P (500 mg/L) was prepared by diluting 12.5 mL 1000 mg/L P standard calibration solution to 25 mL with deionized water. A series of calibration working standards of P for the ISAM were diluted by soil mixture solution. The concentrations of the calibration working standards are listed in Table 4.

As element Re is rare in soil, element Re was selected as an internal standard to minimize errors caused by instrumental drift and was added in situ. A 10 mg/L Re internal standard solution was prepared by diluting 5 mL of a 1000 mg/L Re standard solution to 500 mL with deionized water.

All calibration standards were then stored in the freezer at a temperature of 5°C prior to analysis and were shaken before measurement to ensure proper mixing of P in solutions.

#### 2.4.2. Calibration Working Standards for CCM and CSAM

Calibration working standards of P in the 0–3 mg/L range were prepared by diluting the stock calibration standards with deionized water. Re (10 mg/L) was selected as an internal standard to minimize errors caused by instrumental drift and was added in situ.

All calibration standards were then stored in the freezer at a temperature of 5°C prior to analysis and were shaken before measurement to ensure proper mixing of P in solutions.

### 2.5. Calculation of Detection Limit (*L*_D_) and Quantification Limit (*L*_Q_)

Once the method was set up, the detection limit (*L*_D_) and quantification limit (*L*_Q_) were calculated.

The *L*_D_ was calculated from the measurement of the blank sample. The blank sample was measured ten times under reproducibility conditions. The detection limit was obtained from the following expression [18]:

(1) $$\begin{array}{c} L_{\text{D}}=3.29\;*\;S, \end{array}$$

where *S* = value of standard deviation of the measurements.

The *L*_Q_ was calculated according to the IUPAC guidelines as ten times the standard deviation of the measurement, for a number of the measurements equal to ten [21]:

(2) $$\begin{array}{c} L_{\text{Q}}=10\;*\;S. \end{array}$$

### 2.6. Recovery Test

Duplicate samples of 4 certified standard soils (GSS-5, GSS-8, GSS-14, and GBW (E) 070041) and blank were spiked with 50 *μ*L of a 500 mg/L P standard solution. The spiked samples were digested and measured in the same manner as described in Section 2.3. Run blanks with all the chemicals and process except the soil sample.

Recovery rate was calculated using the following equation:

(3) $$\begin{array}{c} \text{recovery}\;\;\mathrm{rate}=\frac{\left(\left(\text{P}\;\;\mathrm{content}\;\;\mathrm{in}\;\;\mathrm{spiked}\;\;\mathrm{sample}\right)-\left(\text{P}\;\;\mathrm{content}\;\;\mathrm{in}\;\;\mathrm{unspiked}\;\;\mathrm{sample}\right)\right)}{\text{known}\;\;P\;\;\mathrm{content}\;\;\mathrm{added}\;\;\mathrm{to}\;\;\mathrm{spiked}\;\;\mathrm{sample}}\text{.} \end{array}$$

## 3. Results and Discussion

### 3.1. Theory

Generally, the CSAM was used for the determination of total P in soil, and multiple standard curves were obtained as *y*_1_ = *m*_1_*x*_1_ + *b*_1_, *y*_2_ = *m*_2_*x*_2_ + *b*_2_,…, *y*_*n*_ = *m*_*n*_*x*_*n*_ + *b*_*n*_, corresponding to multiple test samples. The content of each test sample can be obtained from the intercept of each standard curve with the X-axis, which was *b*_*n*_/*m*_*n*_. When the matrix of the batch sample was the same or similar, the values of *m* were close to each other and could be approximated as *m*_1_ = *m*_2_ = ⋯ = *m*_*n*_ = *m*. Therefore, for a series of samples with the same or similar matrix, the slopes were assumed to be the same.

In this study, an ISAM based on the CSAM and CCM was proposed. One sample was determined by CSAM. The value of slope, *m*, was calculated from the obtained calibration curve. Then, all the samples were measured directly by ICP-OES, and the response signal values, *b*_*n*,_ were obtained. Hence, the concentration of total P (*C*_*n*_) in the each test sample was calculated according to the following equation:

(4) $$\begin{array}{c} C_{n}=\frac{b_{n}}{m}. \end{array}$$

**Figure 1:**
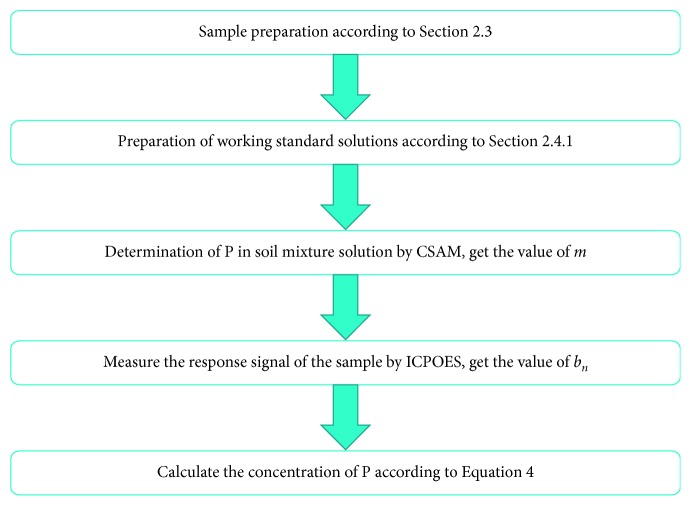
Procedure of analysis by ISAM.

The procedure of analysis by ISAM is shown in Figure 1. Actually, the matrix of the soil samples was not completely the same. To further reduce the error, the soil mixture solution prepared according to Section 2.4.1 was treated as sample and used to obtain the value of *m* using the CSAM.

### 3.2. The Use of Soil Mixture Solution

**Figure 2:**
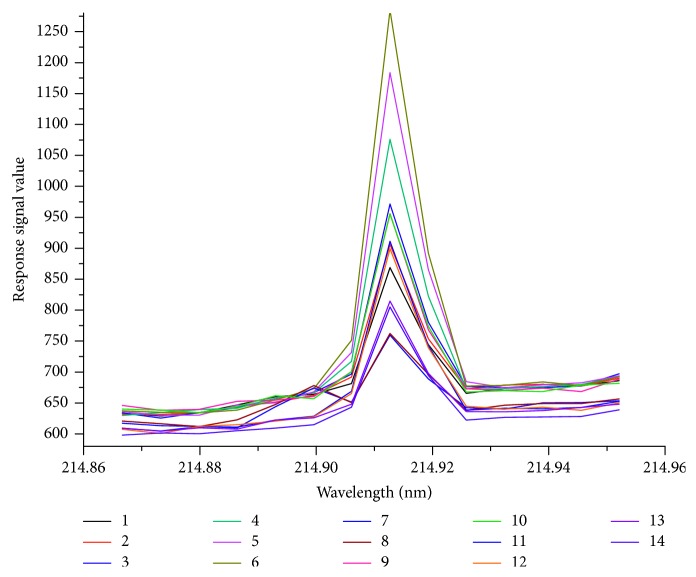
ICP-OES spectrogram of phosphorus with standard solutions diluted by soil mixture solution.

**Figure 3:**
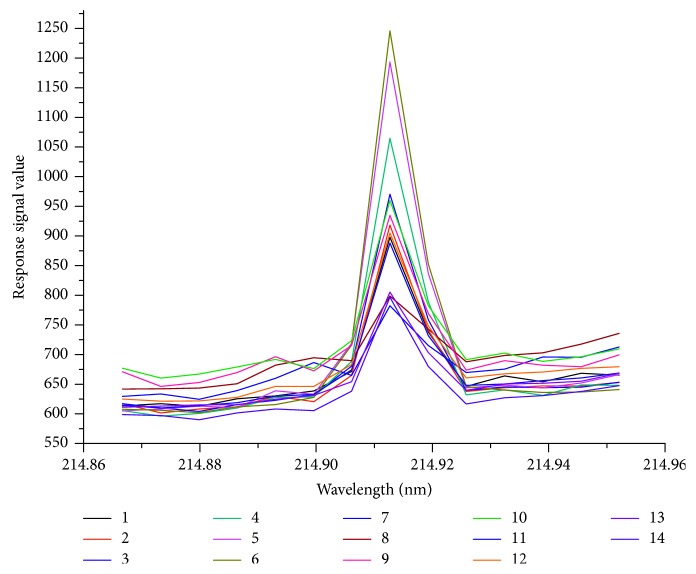
The ICP-OES spectrogram of phosphorus with standard solutions diluted by soil digestion solution of GSS-14.

Total P of the 4 certified standard soils (GSS-5, GSS-8, GSS-14, and GBW (E) 070041) were determined by the proposed ISAM with standard solutions diluted by soil digestion solution of GSS-14 and with standard solutions diluted by soil mixture solution. The corresponding linear correlation relationship for the standard solutions diluted by soil digestion solution of GSS-14 was obtained as *y* = 132.32 (±1.58)*x* + 193.31 (±1.94), *r*=0.9998, and that for the standard solutions diluted by soil mixture solution was obtained as *y* = 95.75 (±1.96) *x* + 108.30 (±1.18), *r* = 0.9997. The results are presented in Table 5, and the ICP-OES spectrograms are shown in Figures 2 and 3.

As shown in Table 5, the differences between the obtained value and certified value (△) were all with the error of the certified value. The average of △_1_ was a little smaller than the average of △_2_. This indicated that the results determined by ISAM with standard solutions diluted by soil mixture solution were more close to the certified values. Thus, to some extent, the use of mixture of soil digestion solution could correct the analytical accuracy.

Operate conditions were as shown in Table 2. Analyte 1⟶6: P standard solution diluted by soil mixture solution with the concentration of 0, 0.2, 0.5, 1.0, 1.5, and 2 mg/L; analyte 7, 8: GSS-5; analyte 9, 10: GSS-8; analyte 11, 12: GSS-14; and analyte 13, 14: GBW (E) 070041.

Operate conditions were as shown in Table 2. Analyte 1⟶6: P standard solution diluted by soil digestion solution of GSS-14 with the concentration of 0, 0.2, 0.5, 1.0, 1.5, and 2 mg/L; analyte 7, 8: GSS-5; analyte 9, 10: GSS-8; analyte 11, 12: GSS-14; and analyte 13, 14: GBW (E) 070041.

Furthermore, as compared to Figure 3, the baselines of the spectrogram in Figure 2 were closer to each other than that in Figure 3. It indicated that the matrix of standard solutions diluted by soil mixture solution was more consistent with the matrix of soil digestion solution. From this perspective, the matrix effect between the standard solution and the digestion solution could be reduced by using the soil mixture solution. Thus, in this study, the soil mixture solution was applied to prepare the calibration working standard solutions.

### 3.3. Determination of Total *P* Value by ISAM and Comparison with CCM and CSAM

According to the principles discussed in Section 3.1, the concentration of P in soil mixture solution was determined by CSAM. The corresponding linear correlation relationship was obtained as follows:

(5) $$\begin{array}{c} y=95.75\left(\pm 1.95\right)x+108.3\left(\pm 1.18\right);\;\;r=0.9997. \end{array}$$

According to Equation (5), the value of *m*, the slope of Equation (5), was 95.75. Taking GSS-5 soil for instance, the digested solution of GSS-5 was measured by ICP-OES directly, and the response signal value (b_GSS-5_) was 75.10. According to Equation (4), the concentration of total P in the GSS-5 digested solution was calculated as *C*_GSS-5_ = *b*_GSS-5_/*m* = 75.10/95.75 = 0.784 mg/L; thus, the content of P was obtained to be 391 mg/kg. The GSS-5 soil sample was measured 3 times in parallel, and the average result of GSS-5 was 396 mg/kg. The contents of P in other certified standard soils (GSS-8, GSS-14, and GBW (E) 070041) were obtained by the same procedure, and the results are listed in Table 6.

As shown in Table 6, the differences between the obtained and certified values (△_2_) were within the error of the certified value, indicating that the proposed method was of acceptable accuracy. Relative standard deviation (RSD) values of 1.23%–3.94% revealed relatively good precision. The ISAM combines the advantages of the CSAM and CCM, and it overcomes the matrix effect to some extent. From this perspective, the ISAM is a useful and potential method for the batch analysis of P content in soil with a complex matrix.

Meanwhile, to further verify the suitability of the improved method for the determination of total P in soil, the P contents of the above certified standard soils were also determined by the CCM and CSAM. All samples were measured 3 times in parallel, and the average results are presented in Table 6. Table 6 shows that all the values obtained by ISAM, CCM, and CSAM agreed with each other. Additionally, △_2_, △_3_, and △_4_ were all within the error of the certified values. And the average of △_2_ is smaller than the average of △_3_ and △_4_. The RSDs of obtained values were all less than 5%.

Furthermore, the determination of the P in 4 certified standard soils using CSAM spent more than 108 min and that using the ISAM only spent nearly 30 min. Compared with CSAM, the proposed ISAM was an simple and time-saving method. Therefore, the proposed ISAM is a rapid and accurate method for the determination of P in batch samples.

### 3.4. Calibration Curve, Detection Limit, and Quantification Limit

The concentration of P in the blank solution was measured 10 times continuously using ICP-OES. The *L*_D_ and *L*_Q_ were calculated as described in Section 2.5.  Table 7 presents the calibration curve, correlation coefficient, *L*_D_, and *L*_Q_.

As shown in Table 7, the *L*_D_ and *L*_Q_ of the ISAM were higher than that achieved by other methodologies. However, be similar to CSAM, the proposed ISAM method could overcome the matrix effect in some extent. On the other side, be similar to CCM, the ISAM is simple and rapid. Thus, the proposed ISAM is suitable for the P determination of the batch sample with matrix effect.

### 3.5. Recovery Test

As described in Section 2.6, the results of the recovery test are listed in Table 8.

As shown in Table 8, the recovery rates of soil samples and the blank were all within the range of 90%–110%. This indicated that the ISAM was acceptable for the determination of the total P content in soil.

### 3.6. Test of Soil Samples

The P content of soil samples from Guangnan County of Yunnan Province, China, and the meadow soil from Qinghai-Tibet Plateau, China, were determined by the proposed ISAM in this report and conventional CCM. The results and relative errors (RE) are presented in Table 9.

The soil samples 1–20 in Table 9 were from Qinghai-Tibet Plateau, China, and the soil samples 21–40 were from Guangnan County of Yunnan Province. As shown in Table 9, the P content of soil samples from Guangnan County of Yunnan Province, China, were higher the that from Qinghai-Tibet Plateau, China. The results from the ISAM and CCM were close to each other, and the RE values between 2 methods were all below 6.23%. The difference of results from the 2 methods was compared with *t*-test. *T*-test showed that the results between ISAM and CCM had no significant difference (*P* > 0.05). Therefore, the improved method ISAM was an acceptable method to determine P content in soil sample.

## 4. Conclusions

A novel method ISAM, based on the CCM and CSAM, was developed to measure the content of total P in soil. The results obtained by the proposed method were compared with the certified values and those determined by conventional methods. It was justified that the ISAM was an acceptable method for evaluation of P content in soil. The developed method, combining the advantages of the CCM and CSAM, overcomes the matrix effect and is suitable for the rapid and accurate analysis of batch samples. The proposed ISAM method is a potential method for the determination of P content in soil and offers a new choice for the determination.

## Figures and Tables

**Table 1:** Difference between each analysis strategy.

Method	Advantage	Disadvantage
CCM	Rapid and simple	Easily affected by fluctuations in operation conditions and the matrix effect
CSAM	Overcome the matrix effect in some extent	Time-consuming, required a complex procedure.
ISAM	Rapid and simple, overcome the matrix effect in some extent	The detection limit and the quantitation limit were higher than the other two methods

**Table 2:** Operating conditions of ICP-OES.

Observation direction	Radial
Wavelengths (nm)	P 214.914, Re 197.248
RF power (KW)	1400
Auxiliary gas flow rate (L/min)	0.5
Atomizer flow rate (L/min)	0.5
Plasma flow rate (L/min)	18
Observation distance (mm)	15
Gas	Argon

**Table 3:** Instrument parameter of microwave digestion.

Step	Ramp time (min)	Hold time (min)	Temperature (°C)	Power (W)
1	10	10	150	1650
2	5	20	175	1650
3	5	30	200	1650

**Table 4:** Concentrations of the calibration working standards used in ISAM.

Element	Volume of soil mixture solution (mL)	Concentration of stock calibration standards (mg/L)	Volume of stock calibration standards (*µ*L)	Concentration of P in a series of calibration standard solutions (mg/L)
P	10	200	10	0.2
		200	25	0.5
		200	50	1.0
		500	30	1.5
		500	40	2.0
		500	60	3.0

**Table 5:** Comparison of the determined results of total P by ISAM with standard solutions diluted by soil digestion solution of GSS-14 and by the soil mixture solution.

Sample	Standard solutions diluted by soil digestion solution of GSS-14		Standard solutions diluted by the soil mixture solution		Certified value
	Total *P* value obtained by ISAM (mg/kg)	△_1_^a^	Total *P* value obtained by ISAM (mg/kg)	△_2_^b^	(mg/kg)
GSS-5	370	20	396	6	390 ± 34
GSS-8	776	1	765	10	775 ± 25
GSS-14	734	4	717	13	730 ± 28
GBW (E) 070041	544	20	530	6	524 ± 26.2

^a^△_1_ = the value obtained by ISAM with the standard solutions diluted by soil digestion solution of GSS-14—certified value; ^b^△_2_ = the value obtained by ISAM with the standard solutions diluted by the soil mixture solution—certified value.

**Table 6:** Comparison of the determined results of ISAM, CCM, and CSAM.

Sample	ISAM			CCM			CSAM			Certified value
	Value (mg/kg)	△_2_	RSD (%)	Value (mg/kg)	△_3_^a^	RSD (%)	Value (mg/kg)	△_4_^b^	RSD (%)	(mg/kg)
GSS-5	396	6	3.94	381	9	3.44	403	13	1.34	390 ± 34
GSS-8	765	10	3.12	768	7	3.22	767	8	1.19	775 ± 25
GSS-14	717	13	1.23	739	9	1.78	745	15	1.25	730 ± 28
GBW(E)070041	530	6	3.85	509	15	3.40	509	15	1.72	524 ± 26.2

^a^△_3_ = the value obtained by CCM—certified value; ^b^△_4_ = the value obtained by CSAM—certified value.

**Table 7:** Detection and quantitation limits for three methods.

Method	Calibration curve	Correlation coefficient	Detection limit	Quantitation limit
ISAM	*y* = 95.75 (±1.95)*x* + 108.3 (±1.18)	0.9996	0.092	0.279
CCM	*y* = 87.92 (±0.65)x − 1.4 (±1.08)	0.9999	0.059	0.180
CSAM	*y* = 218.5 (±2.32)*x* − 3.2 (±0.2.61)	0.9998	0.032	0.097

**Table 8:** Comparison of the recovery rate for the three methods.

Sample	ISAM	CCM	SAM
	Recovery rate (%)	Recovery rate (%)	Recovery rate (%)
GSS-5	97.7	92.4	98.4
GSS-8	105.9	105.7	96.1
GSS-14	100.0	103.2	96.4
GBW (E) 070041	98.5	92.5	102.7
Blank	98.6	100.8	96.0

**Table 9:** Results of soil samples determined by ISAM and CCM.

Sample	Content of P obtained by ISAM (mg/kg)	Content of P obtained by CCM (mg/kg)	RE (%)
1	174	181	−4.14
2	152	146	4.11
3	155	159	−2.83
4	175	173	1.45
5	196	193	1.56
6	171	166	3.02
7	170	169	0.59
8	143	153	−6.23
9	173	176	−1.99
10	202	209	−3.35
11	187	183	2.19
12	182	180	1.11
13	179	182	−1.92
14	168	159	5.99
15	180	188	−4.27
16	136	141	−3.56
17	163	172	−5.23
18	189	184	3.00
19	145	154	−5.54
20	159	169	−5.64
21	402	396	1.44
22	794	780	1.81
23	422	408	3.39
24	474	451	5.12
25	1138	1164	−2.22
26	343	331	3.72
27	385	384	0.17
28	397	391	1.35
29	501	479	4.64
30	448	447	0.19
31	534	507	5.32
32	437	462	−5.49
33	1071	1079	−0.81
34	597	581	2.76
35	632	608	3.94
36	389	391	−0.46
37	374	361	3.65
38	1742	1745	−0.14
39	867	857	1.16
40	489	502	−2.59

## Data Availability

The data used to support the findings of this study are available from the corresponding author upon request.
